# SfDredd, a Novel Initiator Caspase Possessing Activity on Effector Caspase Substrates in *Spodoptera frugiperda*

**DOI:** 10.1371/journal.pone.0151016

**Published:** 2016-03-15

**Authors:** Zhouning Yang, Ke Zhou, Hao Liu, Andong Wu, Long Mei, Qingzhen Liu

**Affiliations:** State Key Laboratory of Virology and Modern Virology Research Center, College of Life Sciences, Wuhan University, Wuhan, People’s Republic of China; Chinese Academy of Sciences, CHINA

## Abstract

Sf9, a cell line derived from *Spodoptera frugiperda*, is an ideal model organism for studying insect apoptosis. The first notable study that attempted to identify the apoptotic pathway in Sf9 was performed in 1997 and included the discovery of Sf-caspase-1, an effector caspase of Sf9. However, it was not until 2013 that the first initiator caspase in Sf9, SfDronc, was discovered, and the apoptotic pathway in Sf9 became clearer. In this study, we report another caspase of Sf9, SfDredd. SfDredd is highly similar to insect initiator caspase Dredd homologs. Experimentally, recombinant SfDredd underwent autocleavage and exhibited different efficiencies in cleavage of synthetic caspase substrates. This was attributed to its caspase activity for the predicted active site mutation blocked the above autocleavage and synthetic caspase substrates cleavage activity. SfDredd was capable of not only cleaving Sf-caspase-1 *in vitro* but also cleaving Sf-caspase-1 and inducing apoptosis when it was co-expressed with Sf-caspase-1 in Sf9 cells. The protein level of SfDredd was increased when Sf9 cells were treated by Actinomycin D, whereas silencing of *SfDredd* reduced apoptosis and Sf-caspase-1 cleavage induced by Actinomycin D treatment. These results clearly indicate that SfDredd functioned as an apoptotic initiator caspase. Apoptosis induced in Sf9 cells by overexpression of SfDredd alone was not as obvious as that induced by SfDronc alone, and the cleavage sites of Sf-caspase-1 for SfDredd and SfDronc are different. In addition, despite sharing a sequence homology with initiator caspases and possessing weak activity on initiator caspase substrates, SfDredd showed strong activity on effector caspase substrates, making it the only insect caspase reported so far functioning similar to human caspase-2 in this aspect. We believe that the discovery of SfDredd, and its different properties from SfDronc, will improve the understanding of apoptosis pathway in Sf9 cells.

## Introduction

Apoptosis is a type of programmed cell death in multi-cellular organisms that is essential for removing unwanted or damaged cells. Apoptosis is also important in tissue development and can act as a defense mechanism [1]. Caspases are a family of cysteine proteases that play important roles in apoptosis. Caspases are classified according to their biological functions and structures into three groups, which include initiator caspases, effector caspases and inflammatory caspases [2–5].

Caspases are synthesized as inactive zymogens (pro-caspases) containing a prodomain, a large subunit and a small subunit [2]. When apoptosis is initiated, pro-caspase is activated by proteolytic cleavage between prodomain and large subunit, and between large and small subunit. The large and small subunits associate with each other to form a heterodimer, and two heterodimers then form a tetramer that acts as an active unit. An effector caspase is activated by an initiator caspase through cleavage of a specific aspartic acid residue. An initiator caspases usually have a long prodomain that contains a caspase recruit domain (CARD) or death effector domain (DED), which can interact with similar motifs on adapter proteins located upstream of the initiator caspase in the apoptotic pathway. Apoptotic signals trigger oligomerization of adaptor proteins. The interaction between oligomerized adaptors and initiator caspases leads to aggregation, autocatalytic cleavage and activation of initiator caspases. Mammalian caspase-8 has two DED domains and is activated through DED domain-interactions with FADD (Fas-associated protein with death domain). Mammalian caspase-9 bears one CARD domain, and it is activated through CARD-CARD interactions between pro-casapse-9 and Apaf-1 (apoptotic protease-activating factor 1).

Apoptosis is widely studied in the insect *Drosophila melanogaster*. Seven caspases have been identified in *Drosophila* [6, 7], including the initiator caspases Dronc, Dredd and Strica [8–10] and the effector caspases Drice, Dcp-1, Damm and Decay [11–14]. Dronc has a long prodomain containing CARD [8], and Dredd has a prodomain that is highly similar to the DEDs of caspase-8 and -10 [9]. *Spodoptera frugiperda*, a pest insect that belongs to the Lepidoptera order, is a model organism for understanding baculovirus replication that is also used to study apoptosis [15]. In particular, the Sf9 cell line derived from *Spodoptera frugiperda* is an ideal system for study apoptosis because it can produce classical apoptotic response and typical apoptotic bodies that are easily observed under a microscope [16–18]. However, the apoptotic pathway in Sf9 has not been completely identified. Since the identification of the effector caspase Sf-caspase-1 from Sf9 cells in 1997 [19], the initiator caspase Sf-caspase-X has been predicated in several reports [20–22] and intensive efforts have been devoted to identifying these initiator caspases in Sf9. In 2013, the initiator caspase SfDronc was identified in Sf9 [23].

Lepidopteran caspases have been identified and classified into 6 clades, which include the putative effector caspases Lep-caspase-1, -2 and -3 and the putative initiator caspases Lep-caspase-5 and -6 [24]. Dronc homologs belong to the Lep-caspase-5 clade, whereas Dredd homologs belong to the Lep-caspase-6 clade [24]. In the present study, we identified a novel initiator caspase, SfDredd, in Sf9. According to the alignment and a phylogenetic analysis, SfDredd shares a high similarity with insect initiator caspase Dredd homologs and belongs to the Lep-caspase-6 clade. Recombinant SfDredd expressed and purified from *Escherichia coli* (*E*. *coli*) could cleave synthetic caspase substrates *in vitro*. Similar to SfDronc, SfDredd could cleave Sf-caspase-1 *in vitro*, but at a site that was different from that for SfDronc. The protein level of SfDredd increased after Actinomycin D treatment, and silencing SfDredd in Sf9 cells reduced apoptosis and Sf-caspase-1 cleavage induced by Actinomycin D treatment. Co-expression of SfDredd and Sf-caspase-1 resulted in increased apoptosis and Sf-caspase-1 cleavage. However, apoptosis of Sf9 cells induced by overexpression of SfDredd alone was not as obvious and required a longer time than that induced by SfDronc, suggesting that a certain threshold should be reached to activate SfDredd, as was reported for caspase-2 and Dredd [9, 25, 26]. The above results indicate that SfDredd functioned as an initiator caspase. Notably, result of caspase activity assay with *E*. *coli* expressed recombinant SfDredd was unexpected, though it shares a sequence homology with the initiator caspase, it exhibited considerably stronger activity on effector caspase substrate DEVD than to all kinds of the initiator caspase substrates tested. Mammalian caspase-2 is the only caspase reported so far that possesses activity on effector caspase substrates and shares a sequence homology with initiator caspases [27–29]. To our knowledge, SfDredd is the only caspase besides human caspase-2, and the first insect caspase, that shows this property.

## Materials and Methods

### Cells

Sf9 cells were obtained from the China Center for Type Culture Collection (CCTCC) (Wuhan, China) and maintained at 27°C in TNM-FH medium (Invitrogen) supplemented with 10% (v/v) heat-inactivated fetal bovine serum (Gibco).

### Antibodies

Monoclonal mouse antibodies against His-tag (Proteintech) and β-actin (Proteintech) were diluted 1:5000 when used in immunoblotting. Polyclonal rabbit antibody against Sf-caspase-1 recognizing the full length and large subunit of Sf-caspase-1, which was a generous gift from Dr. Nor Chejanovsky (Agricultural Research Organization, The Volcani Center, Israel), was diluted 1:1000 when used in immunoblotting. Polyclonal rabbit antibody against SfDredd recognizing the full length and large subunit of SfDredd was prepared by our laboratory using a SfDredd fragment (amino acid residues 347–455) expressed and purified from *E*. *coli* as an antigen.

### Identification and sequencing of SfDredd cDNA

*SfDredd* was initially identified as a partial sequence in a TBLASTN search of the SPODOBASE database (http://bioweb.ensam.inra.fr/spodobase/) of expressed sequence tag (EST) sequences using BmDredd [30], the Dredd homolog in *Bombyx mori*, as a query. The result of the search included a sequence (Accession no. Sf1P14817-5-1) containing an incomplete open reading frame (ORF) with high similarity to BmDredd. Primers for rapid amplification of cDNA ends (RACE) were designed according to the partial sequence obtained for SfDredd (S1 Table). 5’ and 3’ RACE amplifying coding region and untranslated regions (UTRs) of SfDredd were conducted using the SMARTer™ RACE cDNA Amplification Kit (Clontech, CA, USA). Products from 5’ and 3’ RACE reactions were cloned into pCR-II (TA Cloning^®^ Kit; Invitrogen, CA, USA) after purification and then subjected to sequencing. The sequencing results revealed a sequence containing an ORF of 1659 bp flanked by a 5’UTR of 123 bp and a 3’UTR of 231 bp. The ORF was amplified from Sf9 cDNA by PCR and cloned into pCR-II. Plasmids from 5 positive colonies were subjected to sequencing, and the sequencing results confirmed the sequence information of previous sequencing of RACE products.

### GenBank accession number

The sequence of SfDredd obtained from Sf9 cell cDNA was deposited in GenBank under accession number KU668855.

### Construction of plasmids

Plasmid N-His-pET-28a-SfDredd containing an N-terminal His-tag was constructed by cloning the coding region of SfDredd into the *Bam*H I and *Not* I sites of pET-28a. pET-28a-SfDredd-C-His containing a C-terminal His-tag was constructed by cloning the coding region of SfDredd into the *Nco* I and *Xho* I sites of pET-28a. pIE1-SfDredd containing a C-terminal FLAG-tag was constructed by replacing GFP in pIE1GFP with the coding region of SfDredd. pIE1-Sf-caspase-1 containing a C-terminal HA-tag was constructed by replacing GFP and FLAG-tag with the coding region of Sf-caspase-1 and nucleotide sequence of HA-tag. pIE1-SfDredd-C443A, pIE1-SfDronc-C310A and pIE1-Sf-caspase-1-C178A were constructed by introducing point mutations to the corresponding wild type plasmids by overlapping PCR; the primers used in the PCR are listed in S2 Table. The sequences of all plasmids were confirmed by DNA sequencing.

### Recombinant protein purification

BL21 cells containing expression plasmid with or without the ORF for the protein of interest were grown to a concentration of A_600_ = 0.4 in LB containing 50 μg/mL kanamycin, induced by addition of IPTG (isopropyl-β-D-thiogalactopyranoside) at the final concentration of 0.4 mmol/L and then shaken at 20°C for 6 h at 220 rpm. BL21 cells were centrifuged and resuspended in 20 mmol/L imidazole solution containing 1% Triton X-100 and protease inhibitor cocktail tablet (Roche), followed by 180 cycles of sonication for 4 sec with a 6-sec interval between each cycle and a 5-min interval between every 60 cycles. After centrifugation at 15,000 g for 20 min at 4°C, the supernatant was applied to 500 μL Ni-NTA high affinity resin (Genscript) containing gravity column. The resin was washed using a concentration gradient of imidazole (20 mmol/L, 50 mmol/L, 80 mmol/L) and then eluted using 250 mmol/L imidazole. After quality analysis performed with Coomassie blue staining and immunoblotting, purified proteins were stored in -70°C for later use.

### SDS-PAGE and immunoblotting

Purified proteins and prepared cell lysates were mixed with 5 × SDS loading buffer, incubated at 100°C for 5 min and then subjected to SDS-PAGE. Proteins were visualized by Coomassie blue staining or transferred to nitrocellulose membrane (GE) and then subjected to the following steps. Membranes were blocked using 5% milk in TBST for 1 h and then incubated with primary antibody for 1 h. After they were washed with TBST, the membranes were incubated with HRP-conjugated secondary antibody (Thermo) for 1 h. Antibody binding was detected using LAS 4000 (Fujifilm) after incubating the membrane with SuperSignal West Pico chemiluminescent substrate (Millipore).

### Caspase activity assay

The substrate preference of purified recombinant proteins were assayed using 13 fluorogenic synthetic caspase substrates (MP), among them Ac-DEVD-AFC is the optimal substrate of human caspase-3 and caspase-7, Ac-VDVAD-AFC is the optimal substrate of human caspase-2, Ac-AEVD-AFC is the optimal substrate of human caspase-10, Ac-LEHD-AFC is the optimal substrate of human caspase-9, Ac-DMQD-AFC is the optimal substrate of human caspase-3, Ac-IETD-AFC is the optimal substrate of human caspase-8, Ac-WEHD-AFC is the optimal substrate of human caspase-5, Ac-VEID-AFC is the optimal substrate of human caspase-6, Ac-YVAD-AFC is the optimal substrate of human caspase-1, Ac-LETD-AFC is the optimal substrate of human caspase-8, Ac-IEPD-AFC is the optimal substrate of granzyme B, Ac-LEVD-AFC is the optimal substrate of human caspase-4 and Ac-LEED-AFC is the optimal substrate of human caspase-13. The activities of the prepared cell lysates on Ac-DEVD-AFC were also assayed. Briefly, each purified caspase or cell lysate was mixed separately with 20 μmol/L of each fluorogenic substrate in Na-Citrate buffer (50 mmol/L Tris-HCl, 1 mol/L Na-Citrate, 10 mmol/L DTT, 0.05% CHAPS) [31]. Caspase activity was assessed after incubation at 37°C for 30 min. The fluorescence (excitation 405 nm, emission 510 nm) was measured at 37°C every 2 min for 2 h. The resulting data were used to calculate the maximum slope of each curve, and Prism 6 was used to generate the graph.

### Plasmid transfection

Sf9 cells were transfected using Cellfectin II reagent (Invitrogen) according to the protocol provided by the manufacturer. Briefly, Sf9 cells were seeded to a plate with a confluence of approximately 60% with plating medium (15% TNM-FH and 85% unsupplemented Grace). After a 4-h attachment period, a mixture of plasmid and Cellfectin was added, and the cells were maintained at 27°C for the number of hours indicated. Each plate contained 12 wells, and 3.2 μg plasmid and 3.2 μL Cellfectin were used in each well.

### Actinomycin D treatment

Sf9 cells were treated with ActD at a final concentration of 250 ng/mL. Cells were harvested at 6 h after ActD treatment for later analysis.

### Cell lysate preparation

Sf9 cells were collected by a 1,000 g centrifugation for 10 min and subjected to cell lysates preparation using the following procedure. Collected cells were suspended in lysis buffer (200 mmol/L Tris-HCl pH 7.4, 150 mmol/L NaCl, 1 mmol/L EDTA, 1% Triton X-100) containing Complete Mini EDTA-free protease inhibitor cocktail tablet (Roche). After 3 freeze-thaw cycles, cells were centrifuged at 16,300 g for 10 min at 4°C. Supernatants were taken as cell lysates and stored at -80°C for further analysis. Sf9 cells that underwent apoptosis were collected first, and the supernatant was then used to harvest apoptotic bodies using a 15,000 g centrifugation for 15 min at 4°C. Collected cells and apoptotic bodies were combined to prepare cell lysate using the procedure described above.

### Gene silencing by RNA interference (RNAi)

DNA fragments were amplified by PCR using plasmids containing the desired sequences as templates and with both forward and reverse primers with T7 promoters attached to the 5’ end (S3 Table). The amplified DNA fragments were used as templates to generate RNAs using a MEGAscript^®^ T7 Kit (Ambion, Life technologies). RNAs were purified by phenol-chloroform extraction after treatment with TURBO DNase to remove the template DNA. To obtain the dsRNAs, RNA products were heated at 95°C for 2 min and then slowly cooled to room temperature. Qualities and concentrations of dsRNAs were determined by agarose gel electrophoresis and using a NanoDrop instrument (Thermo Fisher Scientific Inc.). To silence the desired genes, 1 μg of dsRNA was transfected into 5×10^5^ Sf9 cells in 12-well plates. dsRNA against *GFP* was used as negative control in this study.

## Results

### Sequence and phylogenetic analysis of SfDredd

**Fig 1:**
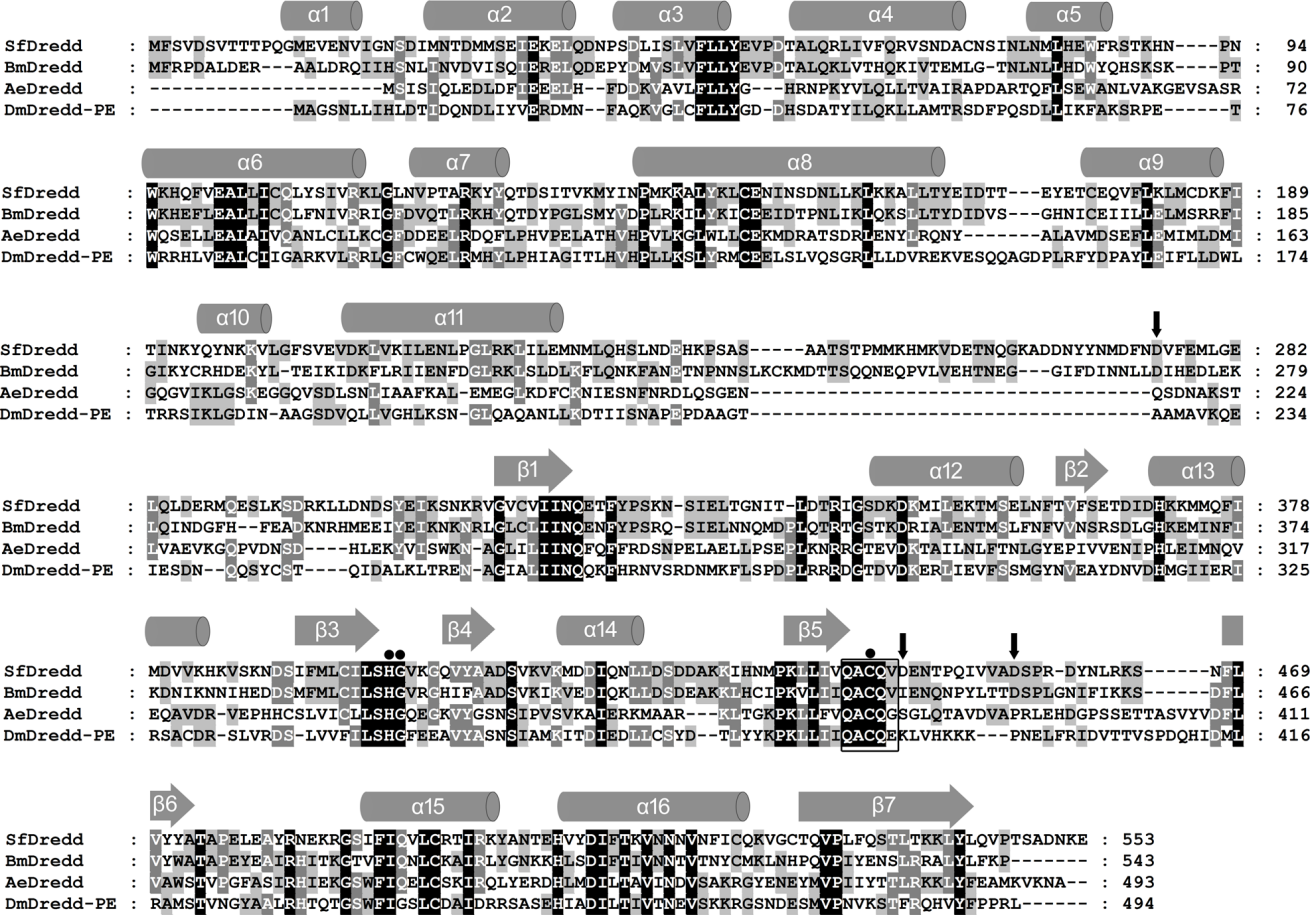
The sequence of SfDredd. The predicted amino acid sequence of SfDredd is shown in alignment with Dredd homologs from *Bombyx mori* (BmDredd), *Aedes aegypti* (AeDredd) and *Drosophila melanogaster* (DmDredd-PE). The amino acid residues identical among 4 Dredds are indicated by white letters within black boxes, the amino acid residues identical among 3 Dredds are indicated by white letters within dark gray boxes, and the amino acid residues identical between 2 Dredds are indicated by black letters within light gray boxes. The alignment was performed using ClustalX 2.1 and modified by GeneDoc 3.2. Secondary structures were predicted using JPred3. Box: catalytic center, closed circles: conserved residues responsible for the catalytic reaction, black arrow: predicted cleavage sites.

Using sequence of BmDredd as a query to search homolog in SPODOBASE [32] (http://bioweb.ensam.inra.fr/spodobase/) by TBLASTN, a sequence (Accession no. Sf1P14817-5-1) highly similar to BmDredd, the Dredd homolog in *Bombyx mori* [30] was identified. Based on that sequence information, a fragment containing a complete ORF of 1659 bp as well as a 5’UTR of 123 bp and a 3’UTR of 231 bp was obtained by 5’ and 3’ RACE reactions. The obtained ORF sequence encodes a putative protein of 552 amino acids (predicted molecular mass of 63 kDa) with a prodomain of 31 kDa, a large subunit of 20 kDa and a small subunit of 12 kDa. The protein shares a high similarity with Dredd homologs (Fig 1) and was therefore named SfDredd. Alignment of the amino acid sequence of SfDredd with the Dredd homologs of *Bombyx mori* (BmDredd), *Aedes aegypti* (AeDredd) and *Drosophila melanogaster* (DmDredd-PE) revealed that SfDredd shared 47%, 25% and 25% amino acid identity with BmDredd, AeDredd and DmDredd, respectively (Fig 1). Putative SfDredd possess the typical caspase catalytic site sequence QACQV with a catalytic cysteine at position 443 (Fig 1). The predicted secondary structure of SfDredd consisted of a series of alpha helices and beta sheets that are highly conserved in other caspases [33] (Fig 1). Three possible cleavage sites were predicted according to the alignment and cleavage sites in other caspases. The predicated cleavage sites included a cleavage site between prodomain and large subunit at D275, and two cleavage sites between the large subunit and small subunit at D446 and D456 with a possible linker between E447 and D456 (Fig 1).

**Fig 2:**
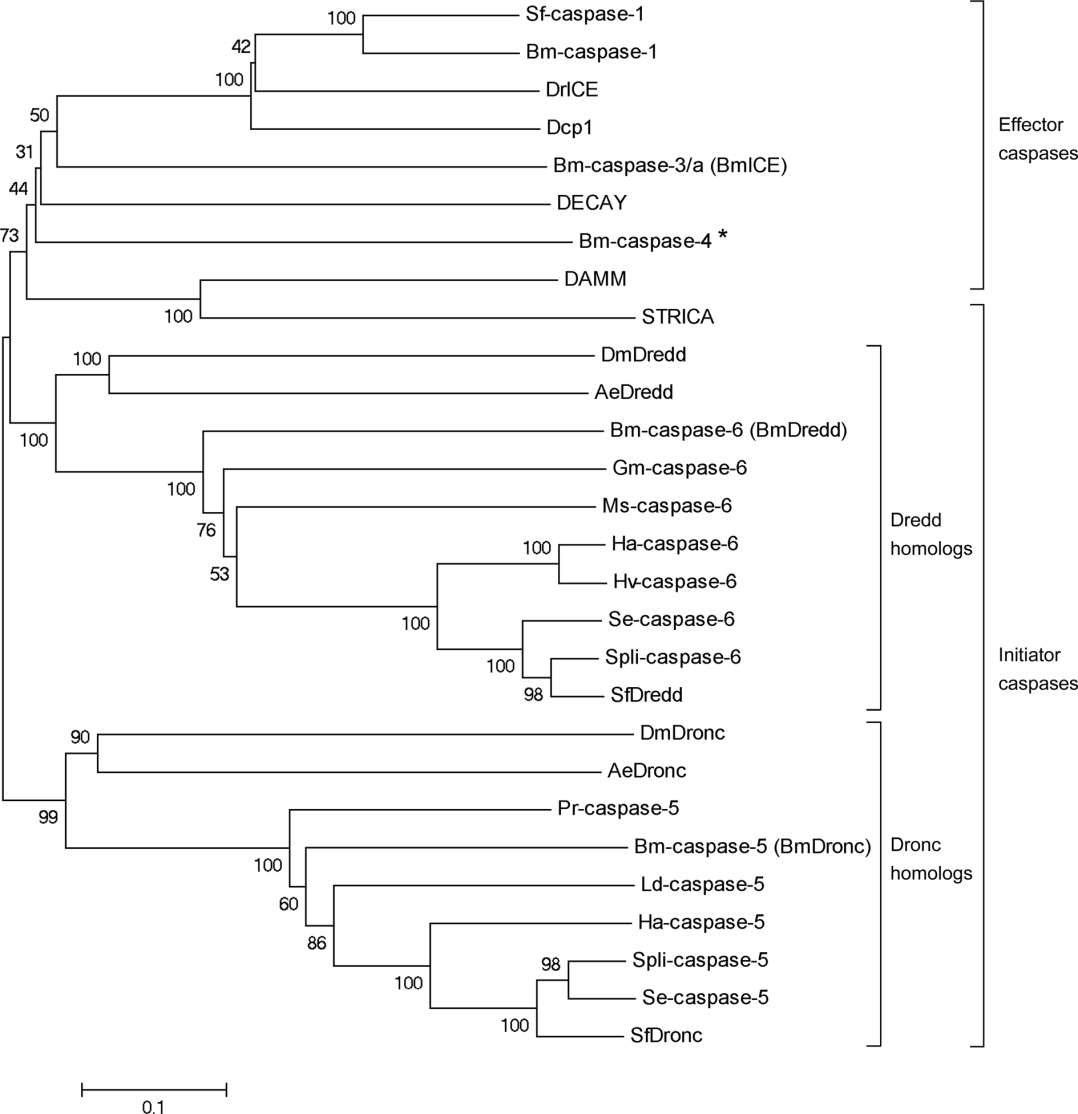
Phylogenetic analysis of SfDredd with selected insect caspases. The predicted amino acid sequence of SfDredd together with 27 selected insect caspases were aligned and the phylogenetic tree was constructed by MEGA 5.05 using the neighbor-joining method. The sequences include the following: SfDredd, SfDronc and Sf-caspase-1 from *Spodoptera frugiperda*, Spli-caspase-5 and Spli-caspase-6 from *Spodoptera litura*, Se-caspase-5 and Se-caspase-6 from *Spodoptera exigua*, Hv-caspase-6 from *Heliothis virescens*, Ha-caspase-5 and Ha-caspase-6 from *Helicoverpa armigera*, Ms-caspase-6 from *Manduca sexta*, Gm-caspase-6 from *Galleria mellonella*, Bm-caspase-1, Bm-caspase-3/a (BmICE), Bm-caspase-4, Bm-caspase-5 (BmDronc) and Bm-caspase-6 (BmDredd) from *Bombyx mori*, Ld-caspase-5 from *Lymantria dispar*, Pr-caspase-5 from *Pieris rapae*, AeDredd and AeDronc from *Aedes aegypti*, Dcp1, DECAY, DAMM, DrICE, STRICA, DmDredd and DmDronc from *Drosophila melanogaster*. Genbank accession numbers of sequences are listed in S4 Table. *: Bm-caspase-4 belongs to Lep-caspase-4 which is a peculiar caspase, whether it belongs to initiator or effector caspase has not been decided [24].

Phylogenetic analyses of SfDredd and 27 selected insect caspases indicated that SfDredd belongs to the Dredd homolog clade, and the closest relatives of SfDredd are Dredd homologs in lepidopteran insects, including caspase-6 in *Spodoptera litura*, *Spodoptera exigua*, *Helicoverpa armigera* and *Heliothis virescens*. Consistent with previously published information [23], SfDronc belongs to the Dronc homolog clade (Fig 2).

### SfDredd underwent autocatalytic cleavage when expressed and purified from *E*. *coli*

**Fig 3:**
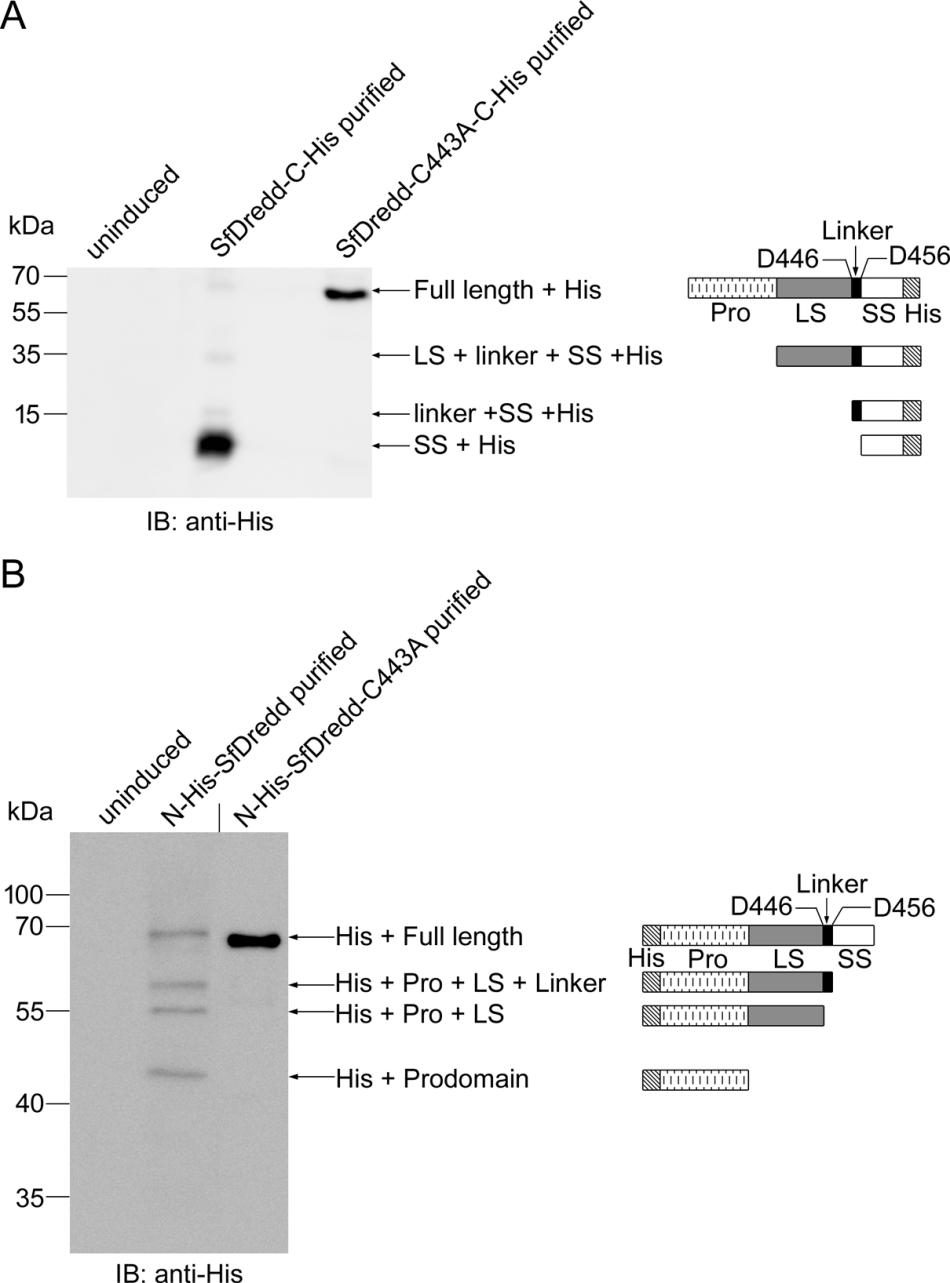
SfDredd underwent autocatalytic cleavage when expressed and purified from *E*. *coli*. C-terminally His-tagged **(A)** or N-terminally His tagged **(B)** SfDredd and their active site mutant SfDredd-C443A were expressed and purified from *E*. *coli* and detected by immunoblotting using antibody against His-tag following SDS-PAGE. Diagrams showing the full length SfDredd and cleaved subunits are on the right. A short vertical black line was used to indicate where lanes were removed and separate parts of the same Western blot image were joined together. His: His-tag, Pro: prodomain, LS: large subunit, L: linker, SS: small subunit.

According to the “induced-proximity” model, caspases often autoprocess themselves when brought into close proximity of each other [34, 35]. Consistent with this, four major bands with molecular masses of approximately 65 kDa, 35 kDa, 15 kDa and 13 kDa were obtained when C-terminally His-tagged SfDredd was expressed and purified from *E*. *coli* and monitored by immunoblotting (Fig 3A). The 65-kDa band matched the C-terminally His-tagged full length of recombinant SfDredd. The 35-kDa band matched the predicted fragment containing the large subunit, linker and small subunit with the C-terminal His-tag (LS + Linker + SS + His). The 15-kDa band matched the predicted fragment containing the linker and small subunit with the C-terminal His-tag (Linker + SS + His). The 13-kDa band matched the predicted fragment containing the small subunit with the C-terminal His-tag (SS + His) (Fig 3A). These cleaved bands suggest SfDredd underwent autocatalytic cleavage when expressed and purified in *E*. *coli*. To confirm the autocleavage properties of SfDredd, the above experiment was repeated with N-terminally His-tagged SfDredd, and autocleavage of SfDredd was also observed (Fig 3B). Four bands matched the predicted N-terminally His-tagged full length (70 kDa); N-terminal His-tag with the prodomain, large subunit and linker (His + Pro + LS + Linker, 57 kDa); N-terminal His-tag with the prodomain and large subunit (His + Pro + LS, 55 kDa) and N-terminal His-tag with the prodomain (His + Prodomain, 45 kDa) were detected (Fig 3B). To study whether the observed autocleavage of SfDredd was caspase activity dependent, the cysteine at 443 (C443) in the predicted active site of SfDredd was mutated to alanine. The mutant SfDredd-C443A was expressed and purified from *E*. *coli* and subjected to immunoblotting. Unlike the wild type SfDredd, the SfDredd active site mutant did not undergo autocleavage; purification of N-terminally or C-terminally His-tagged SfDredd-C443A mutant obtained only one major band matching the full length SfDredd. The above data indicate that SfDredd caspase activity was required for the observed autocatalytic cleavage (Fig 3A and 3B). Thus, taken together, SfDredd possessed caspase activity.

### SfDredd possessed the strongest activity on substrates of effector caspase

**Fig 4:**
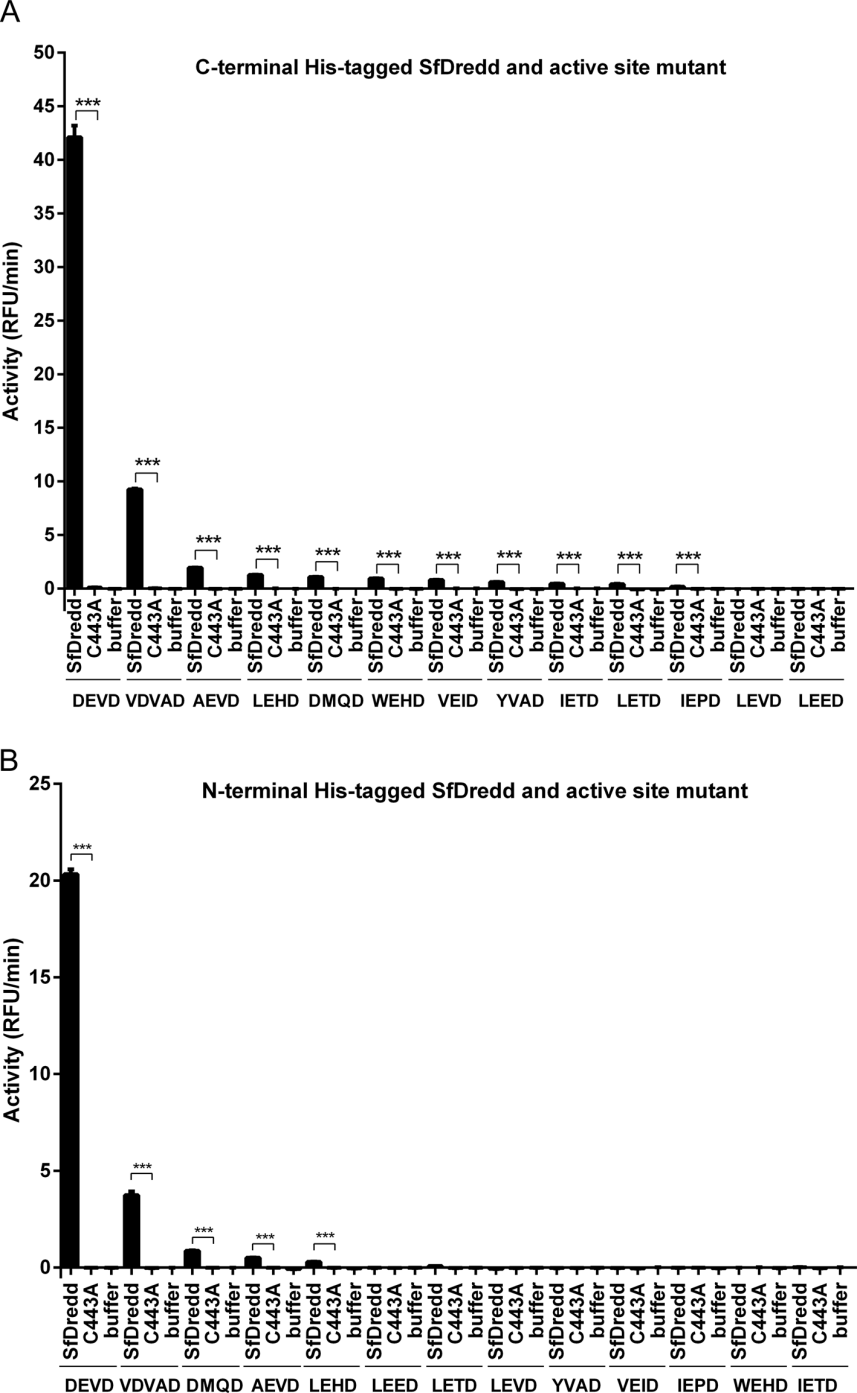
Recombinant SfDredd possessed the strongest activity on effector caspase substrates. Wild type SfDredd and active site mutant SfDredd-C443A with C-terminal His-tag **(A)** or N-terminal His-tag **(B)** were incubated, respectively with 13 types of synthetic caspase substrates (20 μmol/L) and subjected to caspase activity assay. Caspase activity was indicated as the changes in relative fluorescence units (RFU) per minute. The data were presented with the SD from three independent experiments, and statistical significance was calculated by *t* test, ***P < 0.001.

**Fig 5:**
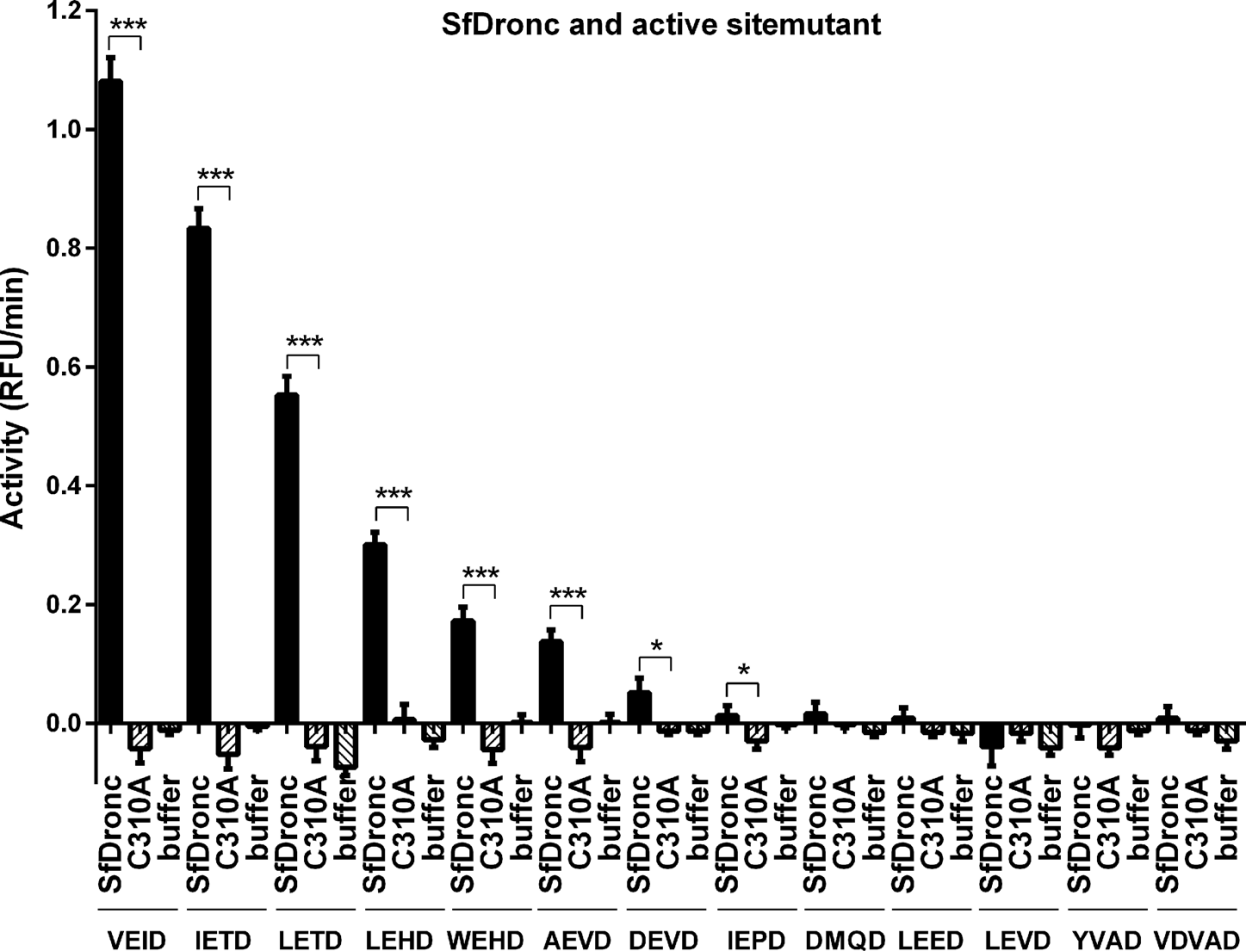
Recombinant SfDronc possessed strongest activity on initiator caspase substrates. Wild type SfDronc and active site mutant SfDronc-C310A with C-terminal His-tag were incubated, respectively with 13 types of synthetic caspase substrates (20 μmol/L) and subjected to caspase activity assay. Caspase activity was indicated as the changes in relative fluorescence units (RFU) per minute. The data were presented with the SD from three independent experiments, and statistical significance was calculated by *t* test, ***P < 0.001, *P < 0.05.

Synthetic caspase substrates are widely used in detecting caspase activity [31, 36]. To confirm that SfDredd indeed possessed caspase activity, enzymatic activities of C-terminally His-tagged SfDredd recombinant protein against 13 different types of synthetic caspase substrates were assayed. Compared with buffer, C-terminally His-tagged SfDredd showed significant activities to Ac-DEVD-AFC, Ac-VDVAD-AFC, Ac-AEVD-AFC, Ac-LEHD-AFC, Ac-DMQD-AFC, Ac-WEHD-AFC, Ac-VEID-AFC, Ac-YVAD-AFC, Ac-IETD-AFC, Ac-LETD-AFC and Ac-IEPD-AFC (Fig 4A), whereas the C-terminally His-tagged active site mutant, SfDredd-C443A, showed no activity on any of the tested substrates. The fact that the caspase active site mutation led to a complete loss of SfDredd activity indicated the activity detected for the wild type SfDredd was conferred by its caspase activity. Surprisingly, despite that SfDredd shares a sequence homology with the initiator caspase, it showed its highest activity on Ac-DEVD-AFC, which is a typical substrate for effector caspase. To confirm the unusual substrate preference of SfDredd, the activity of purified N-terminally His-tagged SfDredd and its active site mutant were also tested, and similar results were obtained. Similar to C-terminally His-tagged SfDredd, the N-terminally His-tagged wild type SfDredd also showed its highest activity to Ac-DEVD-AFC and Ac-VDVAD-AFC, whereas the active site mutant showed no activity on any of the substrates tested (Fig 4B). To further confirm the strong activity of SfDredd to Ac-DEVD-AFC was not an artefact introduced by the experimental system itself, SfDronc, an initiator caspase of *Spodoptera frugiperda* that was described in a recent publication, was expressed, purified from *E*. *coli* and subjected to the caspase activity assays of the 13 synthetic caspase substrates described above. Using the same experimental system, SfDronc showed activity on Ac-VEID-AFC, Ac-IETD-AFC, Ac-LETD-AFC, Ac-LEHD-AFC, Ac-WEHD-AFC or Ac-AEVD-AFC and very low activity on Ac-DEVD-AFC (Fig 5), which is consistent with previously published data [23]. These above data proved that *E*. *coli* expressed recombinant SfDredd indeed possessed the strongest activity on effector caspase substrates.

### SfDredd cleaved Sf-caspase-1 *in vitro* and in Sf9 cells

**Fig 6:**
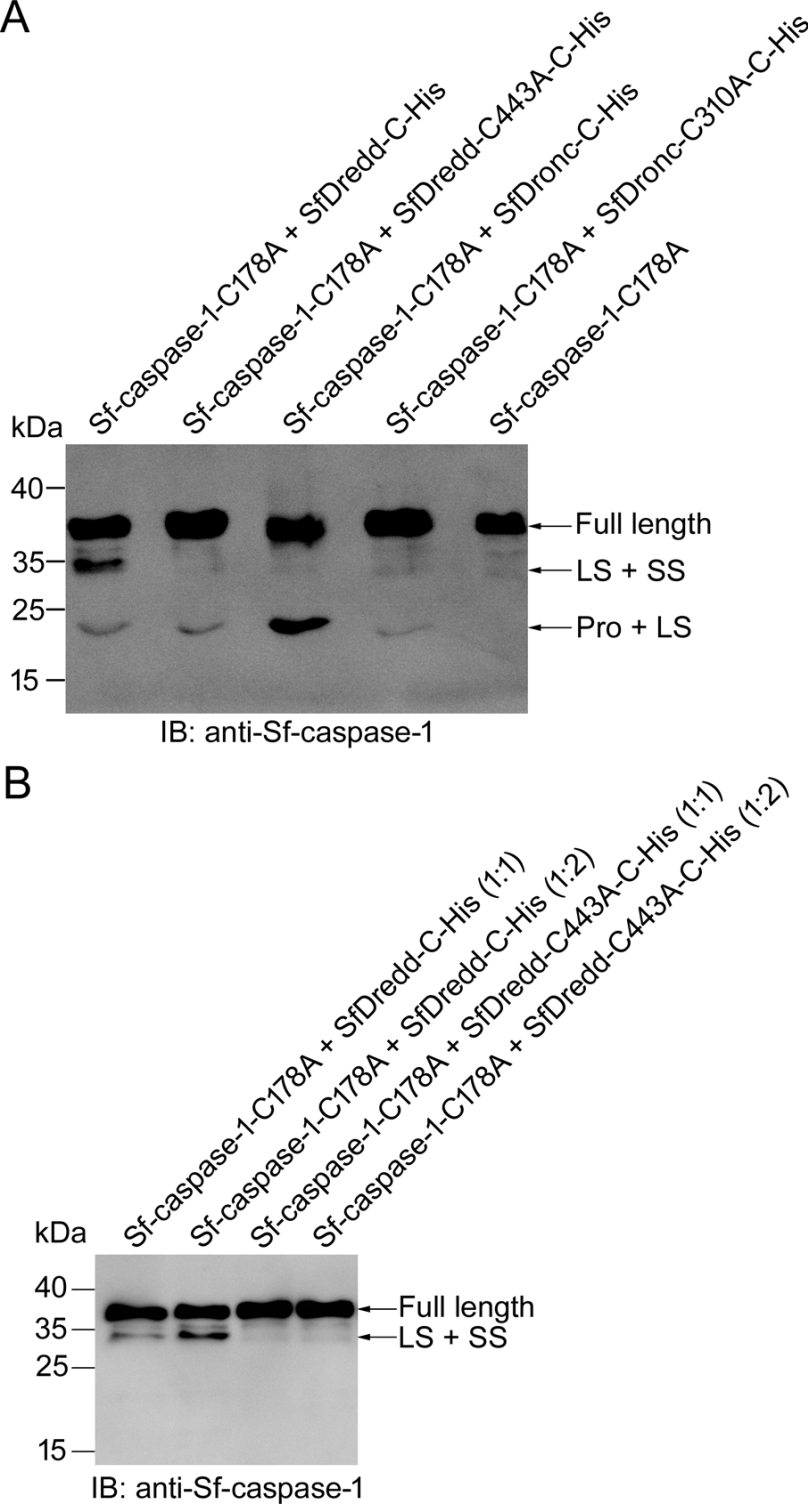
SfDredd directly cleaved Sf-caspase-1 *in vitro*. **(A)** Sf-caspase-1-C178A (300 nmol/L) was incubated with 300 nmol/L of C-terminally His-tagged SfDredd, SfDredd-C443A or SfDronc at 37°C for 1 h in Na-Citrate buffer and then subjected to immunoblotting using an antibody against Sf-caspase-1. **(B)** Sf-caspase-1-C178A (300 nmol/L) was incubated with 300 nmol/L or 600 nmol/L of C-terminally His-tagged SfDredd or SfDredd-C443A at 37°C for 1 h in Na-Citrate buffer and then subjected to immunoblotting using an antibody against Sf-caspase-1.

Because recombinant SfDredd showed the highest activity on Ac-DEVD-AFC, we examined whether SfDredd functioned as an effector caspase or an initiator caspase. We first examined whether SfDredd could cleave Sf-caspase-1, the effector caspase of *Spodoptera frugiperda*, *in vitro*. Recombinant SfDredd and Sf-caspase-1 were incubated at 37°C for 1 h, and cleavage of Sf-caspase-1 was detected by immunoblotting using antibody against Sf-caspase-1. C-terminally His-tagged SfDredd was used in this experiment because it showed stronger caspase activity than N-terminally His-tagged SfDredd (Fig 4). Additionally, to avoid autocatalytic cleavage of Sf-caspase-1, the active site mutant of Sf-caspase-1 (Sf-caspase-1-C178A) was used in this experiment. Sf-caspase-1 was cleaved by SfDredd, but not by its active site mutant (Fig 6A), proving that SfDredd could directly cleave Sf-caspase-1 and the cleavage required the caspase activity of SfDredd. Furthermore, cleavage of Sf-caspase-1 increased when the amount of SfDredd increased (Fig 6B), further confirming the cleavage of Sf-caspase-1 was caused by SfDredd. SfDronc, which was shown to cleave Sf-caspase-1 [23], was used as a positive control in this experiment. As reported, SfDronc could cleave Sf-caspase-1 (Fig 6A). Notably, SfDredd mainly cleaved Sf-caspase-1 at the site between the prodomain and large subunit, whereas SfDronc mainly cleaved the site between the large and small subunits of Sf-caspase-1. This phenomenon is further considered in the discussion.

**Fig 7:**
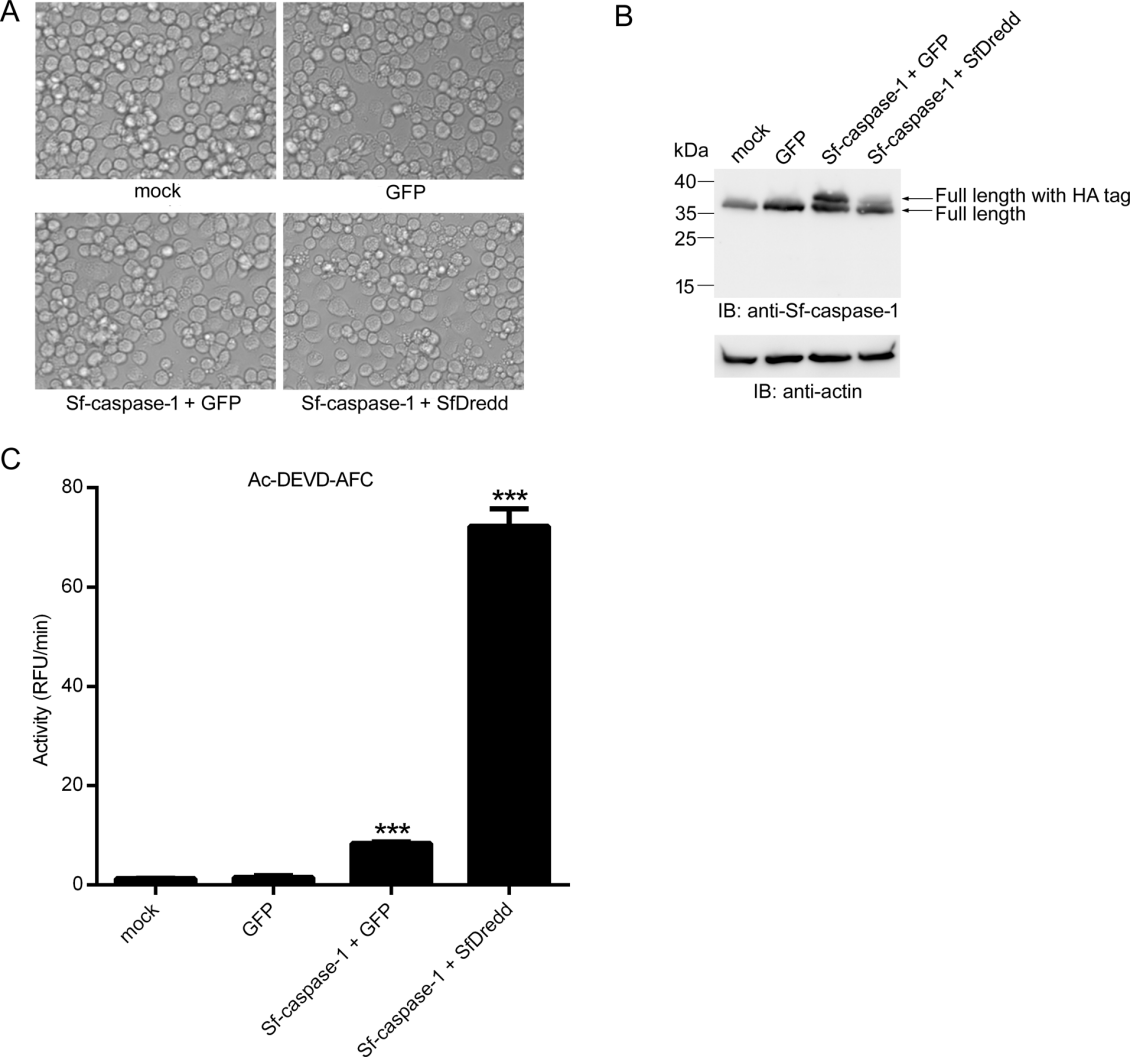
SfDredd cleaved Sf-caspase-1 when transfected in Sf9 cells. Plasmids expressing FLAG-tagged SfDredd and HA-tagged Sf-caspase-1 were co-transfected into Sf9 cells. Mock treated cells, GFP expressed Sf9 cells, and, GFP and Sf-caspase-1 co-expressed Sf9 cells were used as controls. At 42 h post transfection, cells were subjected to the following analyses: **(A)** Photographs of cells were taken under microscope (magnification ×200). **(B)** Cell lysates were prepared and analyzed by immunoblotting using antibody against Sf-caspase-1. **(C)** Cell lysates were incubated with Ac-DEVD-AFC and subjected to caspase activity assay. Caspase activity was indicated as the changes in relative fluorescence units (RFU) per minute. The data were presented with the SD from three independent experiments, and statistical significance was calculated by *t* test, *** represent significant difference with all the other columns, *P* < 0.001.

To investigate whether SfDredd could cleave Sf-caspase-1 in Sf9 cells, SfDredd and Sf-caspase-1 were co-expressed in Sf9, and Sf-caspase-1 cleavage was monitored using immunoblotting. Mock treated Sf9 cells, GFP expressed Sf9 cells, and GFP and Sf-caspase-1 co-expressed Sf9 cells were used as controls. At 42 h post transfection, more apoptotic bodies were observed in Sf9 cells transiently co-expressing Sf-caspase-1 and SfDredd than in Sf9 cells transiently co-expressing Sf-caspase-1 and GFP. At the same time point, Sf9 cells transiently expressing GFP and mock treated Sf9 cells remained normal (Fig 7A). Immunoblotting analysis showed that at 42 h post transfection, cleavage of Sf-caspase-1 occurred more frequently when it was co-expressed with SfDredd than with GFP which was indicated by a reduction in the level of full length Sf-caspase-1 in Sf9 cells co-expressing SfDredd and Sf-caspase-1 compared to that in Sf9 cells co-expressing GFP and Sf-caspase-1 (Fig 7B). The large subunit and intermediate forms of Sf-caspase-1 were hardly detected, which might have resulted from rapid degradation after activation of Sf-caspase-1 in this case [37]. These data proved that SfDredd promoted Sf-caspase-1 cleavage. Further, the cell lysates of the above samples were subjected to enzyme activity assay to Ac-DEVD-AFC. It can be seen that the activity of Sf9 cell lysate of co-expressing Sf-caspase-1 and SfDredd was much higher than that of Sf9 cell lysate of co-expressing Sf-caspase-1 and GFP (Fig 7C). According to the above data, SfDredd is able to induce cleavage of Sf-caspase-1 in Sf9 cells. This suggests that SfDredd plays a role of an initiator caspase in the apoptotic pathway in *Spodoptera frugiperda*.

### SfDredd was involved in ActD-induced apoptosis in Sf9 cells

**Fig 8:**
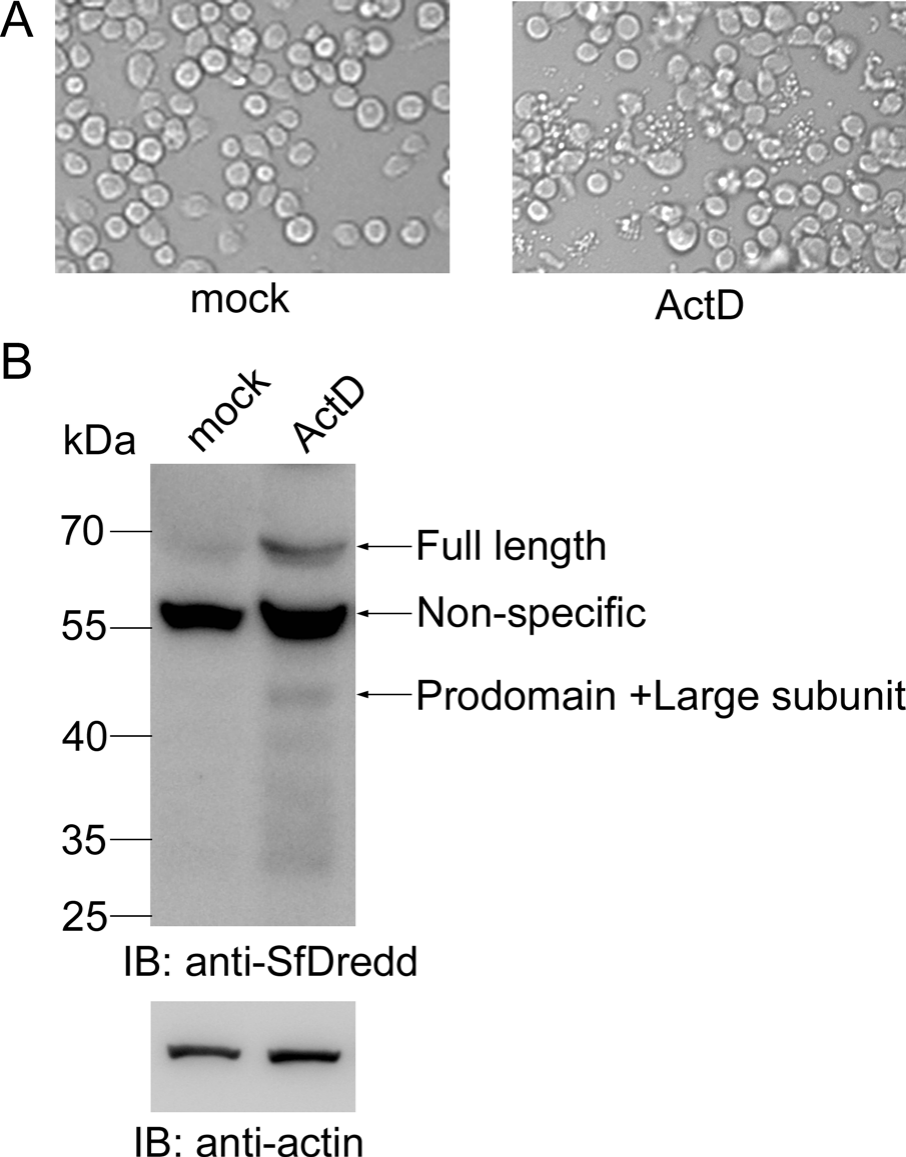
The protein level of SfDredd increased in ActD-treated Sf9 cells. Sf9 cells were treated with ActD at a final concentration of 250 ng/mL. **(A)** Cell pictures were taken 6 h post ActD treatment (magnification ×200). **(B)** Sf9 cells were harvested 6 h after ActD (250 ng/mL) treatment, and cell lysates were analyzed by immunoblotting using antibody against SfDredd.

To study the function of SfDredd in the apoptosis pathway in Sf9 cells, we treated Sf9 cells with the apoptosis stimulus ActD and measured the protein level of SfDredd using immunoblotting. Apoptotic bodies were produced 6 h after ActD treatment, thus indicating apoptosis of Sf9 cells (Fig 8A), and the protein level of SfDredd was significantly increased (Fig 8B). This result suggests that SfDredd was involved in ActD-induced apoptosis in Sf9 cells.

**Fig 9:**
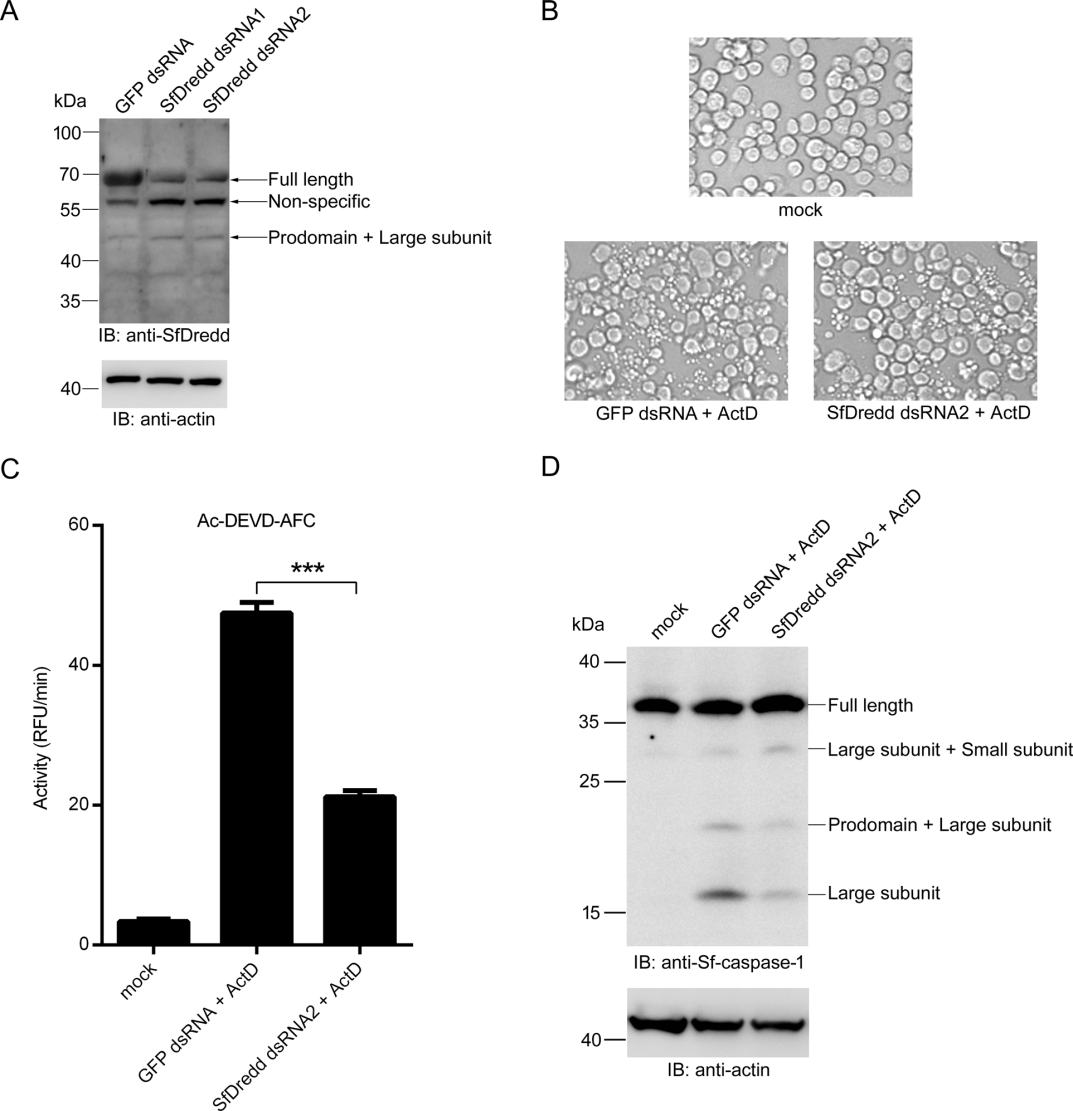
Knockdown of *SfDredd* led to decreased apoptosis induced by ActD. **(A)** 5×10^5^ Sf9 cells were transfected with dsRNA (1 μg) of *SfDredd*-dsRNA or *GFP*-dsRNA, 24 h later, cells were harvested and subjected to immunoblotting using antibody against SfDredd. **(B-D)** 5×10^5^ Sf9 cells were transfected with dsRNA (1 μg) of *SfDredd*-dsRNA or *GFP*-dsRNA, 24 h later, cells were treated with ActD at a final concentration of 250 ng/mL for 6 h and subjected to the following analyses: (B) Cell images were taken under a microscope (magnification ×200). (C) Cell lysates were incubated with Ac-DEVD-AFC and subjected to caspase activity assay. Caspase activity was indicated as the changes in relative fluorescence units (RFU) per minute. The data were presented with the SD from three independent experiments, and statistical significance was calculated by *t* test, ***P < 0.001. (D) The cleavage of Sf-caspase-1 was detected by immunoblotting using an antibody against Sf-caspase-1.

To confirm the role of SfDredd in apoptosis in Sf9 cells, *SfDredd* expression was knocked down by dsRNA, and the effect on apoptosis was studied. To that purpose, Sf9 cells were transfected with *SfDredd*-dsRNA for 24 h, which led to a partial knock down of *SfDredd* (Fig 9A), and then treated with ActD (Sf9 cells transfected with *GFP*-dsRNA were used as a control). As shown in Fig 9A, *SfDredd* dsRNA2 was more effective in knocking down *SfDredd* expression and thus was used in the experiment. 6 h after ActD treatment, ActD-induced apoptosis was partially inhibited by the *SfDredd* knock down, which was indicated by a smaller number of apoptotic bodies in *SfDredd*-dsRNA treated Sf9 cells than that in *GFP*-dsRNA-treated Sf9 control cells (Fig 9B). Consistent with this finding, knock down of *SfDredd* decreased caspase activity induced by ActD in Sf9 cells (Fig 9C). It is known that ActD treatment results in cleavage of Sf-caspase-1 [20, 38]. Compared with *GFP*-dsRNA treatment, *SfDredd*-dsRNA treatment decreased ActD-induced cleavage of Sf-caspase-1 in Sf9 cells (Fig 9D). These data prove that SfDredd was involved in ActD-induced apoptosis in Sf9 cells.

### Transient expression of SfDredd induced slight apoptosis in Sf9 cells

**Fig 10:**
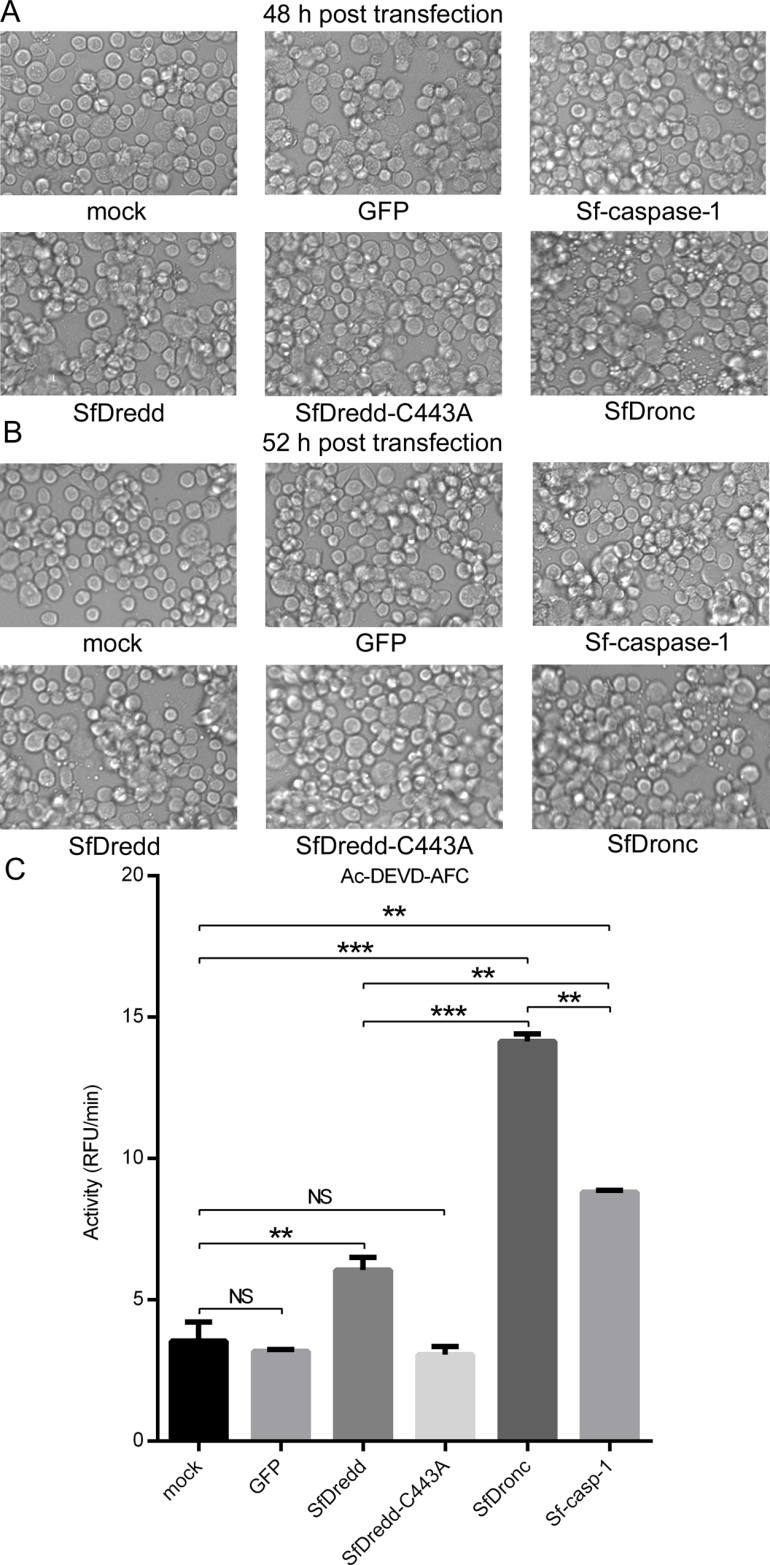
Transient expression of SfDredd induced slight apoptosis in Sf9 cells. Plasmids expressing C-terminally FLAG-tagged SfDredd, active site mutant SfDredd-C443A, Sf-caspase-1, SfDronc and GFP were transfected into Sf9 cells separately. **(A)** Cell pictures were taken 48 h post transfection (magnification ×200). **(B)** Cell pictures were taken 52 h post transfection (magnification ×200). **(C)** Cells were harvested 52 h post transfection and subjected to caspase activity assay. Caspase activity was indicated as the change in relative fluorescence units (RFU) per minute. The data were presented with the SD from three independent experiments, and statistical significance was calculated by *t* test, NS—not significant (*P*>0.05), ** *P*<0.01, *** *P* < 0.001.

Because SfDredd played a role in ActD-induced apoptosis, we examined whether high expression of SfDredd would lead to apoptosis. For this experiment, plasmids containing C-terminally FLAG-tagged SfDredd and its active site mutant, SfDredd-C443A, were transiently transfected into Sf9 cells, and the occurrence of apoptosis was investigated. Mock-treated Sf9 cells, Sf9 cells transfected with SfDronc, Sf-caspase-1 or GFP were used as controls. Different from SfDronc-transfected Sf9 cells showing obvious apoptosis at 48 h and 52 h post transfection [23], apoptosis was not observed in SfDredd transfected Sf9 cells at 48 h post transfection (Fig 10A). Only a small amount of apoptosis was observed at 52 h post transfection (Fig 10B), and the level was similar to that observed in Sf-caspase-1 transfected Sf9 cells. In the caspase activity assay, Sf9 cells that transiently expressed SfDredd showed lower caspase activity than Sf9 cells that transiently expressed SfDronc (Fig 10C), which confirms the result described above. Taken together, these findings indicate that SfDredd might not be the primary apoptotic initiator caspase in Sf9 cells and functioned only when its level reached a threshold, as was reported for caspase-2 and Dredd [9, 25, 26].

## Discussion

In this study, we identified a novel *Spodoptera frugiperda* caspase, SfDredd. This is the second initiator caspase reported in Sf9 cells. Synthetic caspase substrates are widely used in detecting caspase activity and distinguishing between initiator caspases and effector caspases [31, 36]. Although SfDredd shares sequence homology with initiator caspases and functioned as an apoptotic initiator caspase, purified SfDredd exhibited the strongest activity on effector caspase substrates and exhibited weak activity on initiator caspase substrates. To date, human caspase-2 is the only reported caspase that shares sequence homology with initiator caspases while exhibiting effector caspase substrate specificity [27–29]. To our knowledge, SfDredd is the only caspase other than caspase-2, and the first insect caspase, that has been reported to show this property.

The first initiator caspase identified in Sf9 cells was SfDronc, which was reported in 2013 [23]. Our data showed that apoptosis induced in Sf9 cells by SfDredd overexpression alone was less obvious and required a longer time than that induced by SfDronc, suggesting that a certain threshold should be reached to activate SfDredd, as was previously reported for caspase-2 and Dredd [9, 25, 26]. Additionally, the cleavage sites of Sf-caspase-1 by SfDredd and SfDronc are different. Unlike SfDronc that cleaves Sf-caspase-1 mainly at the site between the large and small subunits (D195), SfDredd cleaved Sf-caspase-1 mainly at the site between the prodomain and large subunit (D28). It was reported that Sf-caspase-1 is typically cleaved first at D28 in baculovirus-infection-induced apoptosis, but it was typically cleaved first at D195 in UV-irradiation-induced apoptosis [38]. Thus, we hypothesize that SfDredd and SfDronc may respond to different apoptosis stimuli in *Spodoptera frugiperda*. Our study provides a step forward in completely identifying the apoptotic pathway in *Spodoptera frugiperda*.

## Supporting Information

S1 Table Primers used for RACE. (DOCX)

S2 Table Primers used for mutagenesis. (DOCX)

S3 Table Primers used for dsRNA synthesis. (DOCX)

S4 Table Sequences used for alignments and phylogenetic tree. (PDF)
