# Supplementary material for: SfDredd, a Novel Initiator Caspase Possessing Activity on Effector Caspase Substrates in Spodoptera frugiperda
Source: PLoS One. 2016 Mar 15;11(3):e0151016. doi: 10.1371/journal.pone.0151016 (PMC4792459; doi:10.1371/journal.pone.0151016)
Supplement: S1 Table — (DOCX) [file pone.0151016.s001.docx]

**S1 Table. Primers used for RACE.**

| Primer name | Primer sequence |
| --- | --- |
| RACE-5’-1 | CAGTTGACAGATAAGGAGAGCTTC |
| RACE-3’-4 | CTTCTTTATGAAGTACCGGACACAGC |
