# Supplementary material for: SfDredd, a Novel Initiator Caspase Possessing Activity on Effector Caspase Substrates in Spodoptera frugiperda
Source: PLoS One. 2016 Mar 15;11(3):e0151016. doi: 10.1371/journal.pone.0151016 (PMC4792459; doi:10.1371/journal.pone.0151016)
Supplement: S2 Table — (DOCX) [file pone.0151016.s002.docx]

**S2 Table. Primers used for mutagenesis.**

| Primer name | Primer sequence |
| --- | --- |
| SfDredd-D456A-F | ATTGTAGTGGCTGCCAGCCCAAGAG |
| SfDredd-D456A-R | CTCTTGGGCTGGCAGCCACTACAAT |
| SfDredd-C443A-F | GATAGTTCAAGCAGCCCAAGTTGATG |
| SfDredd-C443A-R  Sf-caspase-1-C178A-F  Sf-caspase-1-C178A-R | CATCAACTTGGGCTGCTTGAACTATC  GTCACCTTGGCAAGCCTGAATAA  TTATTCAGGCTTGCCAAGGTGAC |
