# Supplementary material for: SfDredd, a Novel Initiator Caspase Possessing Activity on Effector Caspase Substrates in Spodoptera frugiperda
Source: PLoS One. 2016 Mar 15;11(3):e0151016. doi: 10.1371/journal.pone.0151016 (PMC4792459; doi:10.1371/journal.pone.0151016)
Supplement: S3 Table — (DOCX) [file pone.0151016.s003.docx]

**S3 Table. Primers used for dsRNA synthesis.**

| Primer name | Primer sequence |
| --- | --- |
| T7-dsSfDredd-1-F  T7-dsSfDredd-1-R | TAATACGACTCACTATAGGGTCCAACGATGCCTGTAACTCTA  TAATACGACTCACTATAGGGTCCAACTGTAACTCTCCAAGCA |
| T7-dsSfDredd-2-F | TAATACGACTCACTATAGGGCTTGACTCTGATGATGCCAAAA |
| T7-dsSfDredd-2-R | TAATACGACTCACTATAGGGCACTGGTGGGCACTTGTAAATA |
| T7-dsEGFP-1-F  T7-dsEGFP-1-R | TAATACGACTCACTATAGGGATGGTGAGCAAGGGCGAGGA  TAATACGACTCACTATAGGGTTGAAGTTCACCTTGATGCC |
