# Supplementary material for: SfDredd, a Novel Initiator Caspase Possessing Activity on Effector Caspase Substrates in Spodoptera frugiperda
Source: PLoS One. 2016 Mar 15;11(3):e0151016. doi: 10.1371/journal.pone.0151016 (PMC4792459; doi:10.1371/journal.pone.0151016)
Supplement: S4 Table — (PDF) [file pone.0151016.s004.pdf]

S4 Table. Sequences used for alignments and phylogenetic tree.

Species	Name	Length	Genbank accession
Aedes aegypti	AeDredd	493 aa	ABI74776.1
Aedes aegypti	AeDronc	449 aa	XP_001655433.1
Bombyx mori	Bm-caspase-1	293 aa	NP_001037050.1
Bombyx mori	Bm-caspase-3/a (BmICE)	284 aa	ABC94941.1
Bombyx mori	Bm-caspase-4	497 aa	AEK71902.1
Bombyx mori	Bm-caspase-5 (BmDronc)	438 aa	NP_001182396.1
Bombyx mori	Bm-caspase-6 (BmDredd)	543 aa	BAF98475.1
Drosophila melanogaster	Dcp1	323 aa	NP_476974.1
Drosophila melanogaster	DECAY	308 aa	AAD54071.2
Drosophila melanogaster	DAMM	255 aa	AAF58613.3
Drosophila melanogaster	DrICE	339 aa	CAA72937.1
Drosophila melanogaster	STRICA (Dream)	527 aa	AAF57292.2
Drosophila melanogaster	DmDredd	494 aa	AAC33117.1
Drosophila melanogaster	DmDronc	450 aa	AAD26625.1
Galleria mellonella	Gm-caspase-6	537 aa	AEK20837.1
Helicoverpa armigera	Ha-caspase-5	453 aa	AEK20835.1
Helicoverpa armigera	Ha-caspase-6	542 aa	AEK20838.1
Heliothis virescens	Hv-caspase-6	547 aa	HQ328982.1
Lymantria dispar	Ld-caspase-5	443 aa	BAL60586.1
Manduca sexta	Ms-caspase-6	543 aa	AEF30497.1
Pieris rapae	Pr-caspase-5 (partial)	252 aa	AEK20836.1
Spodoptera exigua	Se-caspase-5	453 aa	AFX60235.1
Spodoptera exigua	Se-caspase-6	548 aa	AFO64608.1
Spodoptera frugiperda	Sf-caspase-1	299 aa	AAC47442.1
Spodoptera frugiperda	SfDronc	447 aa	JX912275
Spodoptera litura	Spli-caspase-5	445 aa	AFJ04535.1
Spodoptera litura	Spli-caspase-6	522 aa	AFJ04536.1
